# Does fast sintering affect the optical properties, fracture strength, and microstructure of monolithic zirconia?

**DOI:** 10.1007/s10266-025-01110-8

**Published:** 2025-04-28

**Authors:** Mustafa Ayata, Haydar Albayrak, Ravza Eraslan

**Affiliations:** 1 Private Practice, Ortoperio Oral and Dental Health Polyclinic, Kocasinan, Kayseri, Türkiye; 2 Department of Prosthodontics, Faculty of Dentistry, Erciyes University, Melikgazi, Kayseri, Türkiye

**Keywords:** Translucency, Opalescence, Fluorescence, Grain size, Hydrothermal aging, Phase and chemical composition

## Abstract

The purpose of this study was to examine the influence of fast sintering on the optical properties, fracture strength, and microstructure of monolithic zirconia (MZ) ceramics. After 20 disc-shaped and 4 square-shaped samples from the MZ block were machined in the CAM device, groups were created by the sintering procedure in accordance with the manufacturer's recommendations: Standard (Grup S; disc-shaped *n* = 10 square-shaped *n* = 2) and fast (Grup F; disc-shaped *n* = 10 square-shaped *n* = 2). Then, hydrothermal aging was applied to all samples. To calculate translucency, opalescence, and fluorescence values, the LabCh color data of the samples were defined using spectrophotometers. The disc samples were biaxially fracture tested, and the values were determined. The chemical content, grain size (GS), and phase composition of the square-shaped samples were analyzed, respectively. Since the grain size did not conform to the normal distribution, the Mann–Whitney *U* test was used for statistical analysis, and all other variables were analyzed by an independent sample *t* test (*α* = 0.05). The sintering procedure affected translucency, opalescence, fracture strength, and GS significantly (*P* < 0.05), but not fluorescence (*P* > 0.05). While both groups had similar crystal structures, there were some minor differences in chemical content. All samples had a higher fracture strength than the ISO 6872 standard recommends. The sintering procedure had no clinically significant effect on the optical properties of MZ. The fast sintering procedure of MZ can be recommended.

## Introduction

Monolithic zirconia (MZ) restorations have become popular due to their acceptable aesthetic results, their ability to be applied with more conservative tooth preparation, their favorable fracture strength, and the shorter and less laborious laboratory and clinical process [1–3]. Clinical studies have also shown that dental restorations made of zirconia can last a long time [4, 5]. Mechanical properties such as fracture strength are of critical importance in the preference of restorative materials. Studies examining survival rates have revealed that MZ restorations may be prone to fracture due to repetitive occlusal forces [4, 6]. Therefore, it is significant to appraise the fracture strength of MZ restorations in vitro [7]. The biaxial fracture strength (BFS) test is a dependable method proposed to appraise the mechanical limits of materials such as MZ [8].

In addition to the mechanical features of restorative materials, the compatibility of optical properties, such as translucency, opalescence, and fluorescence with natural teeth, is also of critical importance. The translucency parameter (TP) is a dependable procedure for determining the translucency of restorative materials which has been used in numerous studies [9–11]. The TP of restorative materials can be calculated by determining the difference between the color values measured on a black and white background [12]. The enamel of natural teeth has opalescent properties, and mimicking this property in dental restorations is important for aesthetic success [13]. The opalescence parameter (OP) of a restorative material can be computed from the difference between the color values defined under a black and white background [14, 15]. Another parameter that affects the optical appearance of a restoration in the oral cavity is fluorescence. Natural teeth come in sight lighter and whiter under daylight and have a bluish-white fluorescence when exposed to ultraviolet (UV) light [16]. Esthetic dental materials should exhibit fluorescence. The fluorescence parameter (FL) of a dental material can be defined by the color distinction in the presence and absence of UV light. This is measured using a spectrophotometer [17].

MZ restorations are sintered to gain final strength and density [14, 18]; slow, standard, fast, and super-fast sintering processes for different brands of MZs. Fast sintering procedures have made MZ restorations a material that can be finished in a single session [19, 20]. However, different sintering processes can alter the chemical composition [21, 22] and grain size (GS) [10, 14] of MZ, thereby influencing its optical and mechanical properties [23]. In previous studies examining MZ, the fast sintering process was reported to increase [18] or decrease [10] TP values compared to standard sintering. Other previous studies also showed that the OP value of the standard sintering process was lower than the fast sintering process [24] or that the OP value was not affected by the sintering procedure [9, 14]. Previous studies evaluating the fracture strength of MZ with different sintering protocols also reported that the fast sintering either statistically increased [23] or did not affect [24] the fracture strength. For this reason, the influence of sintering on OP, TP, and fracture strength is yet contentious. In addition, only one study was found which investigate the influence of sintering process on the fluorescence feature of MZ [21]. Despite the fact that manufacturers evaluate their sintering protocols before launching the material on the market, independent validation of these protocols is essential for clinical reliability. Manufacturer-reported data are often derived under strictly controlled laboratory conditions that may not reflect clinical scenarios. Furthermore, there are a limited number of studies that simultaneously evaluate the optical features, fracture strength, and microstructure of MZ following hydrothermal aging with both standard and fast sintering [25]. While manufacturers provide guidelines for their recommended sintering protocols, the impact of these protocols under hydrothermal aging conditions remains unclear.

The purpose of this study was to examine the influence of fast sintering on the optical features, fracture strength, and microstructure of MZ ceramics. The null hypothesis of the study was that different sintering procedures would not have a significant effect on the translucency, opalescence, fluorescence, fracture strength, and grain size of MZ ceramic.

## Materials and methods

A disc-shaped sample with a thickness of 1.2 mm and a diameter of 12 mm and a square-shaped (10 × 10 × 2 mm) sample were designed in a three-dimensional design program (SolidWorks Student Edition 2018, France) and stored in the standard tessellation language (STL) file type. Using STL files and a presinterized 3Y-TZP zirconia block (Table 1), 20 disc-shaped and 4 square-shaped samples were machined in a CAM device (Yenadent D40; Yenadent, ZenoTec, Istanbul, Turkey). The calibration of the CAM device was completed before the machining process. Additionally, new milling burs were placed in the CAM device before machining the samples. Disc-shaped and square-shaped samples were randomly (www.random.org) divided into groups, and groups were created by the sintering method according to the manufacturer's recommendation as standard (Grup S; disc-shaped *n* = 10 square-shaped *n* = 2) and fast (Grup F; disc-shaped *n* = 10 square-shaped *n* = 2) procedures (Tables 2, 3). After the sintering process (Programat S1 1600, Ivoclar Vivadent), the last measurements of the samples were verified with a digital caliper (293-821-30, Mitutoyo Corporation, Japan) (± 0.05 mm). The present study excluded any discs with inappropriate dimensions, and new discs were subsequently manufactured.

**Table 1:** Information about the used zirconia

Zenostar T/Wieland Dental/Pforzheim Germany		
Chemical composition	Oxide percentages	Batch code
Zirconium oxide (ZrO_2_ + HfO_2_ + Y_2_O_3_)	≥ 99.0%	U08749
Yttrium oxide (Y_2_O_3_)	> 4.5– ≤ 6.0%	
Hafnium oxide (HfO_2_)	≤ 5.0%	
Aluminium oxide (Al_2_O_3_)	≤ 1.0%	
Type/class (ISO 6872:2015)	Type II/class 5

**Table 2:** Manufacturer's recommended standard sintering procedure

	Temperature 1 °C	Temperature 2 °C	Heating rate: (°C/h)/(°C/min)	Holding time (h)
Heating	20	900	600/10	–
Holding	900	900	–	0.5
Heating	900	1450	200/3.33	–
Holding	1450	1450	–	2
Cooling	1450	900	600/10	–
Cooling	900	300	500/8.33	–

**Table 3:** Manufacturer's recommended fast sintering procedure

	Temperature 1 °C	Temperature 2 °C	Heating rate: (°C/h)/(°C/min)	Holding time (h)
Heating	20	1520	1500/25	–
Holding	1520	1520	–	0.5
Cooling	1520	300	800/13.33	–

Following the sintering process, all samples were put in a sterilization bundle tagged with the sintering process and sample number. Total samples underwent hydrothermal aging in an autoclave [26] (Lina, W&H Sterilization, Italy) at 0.2 MPa pressure, 134 °C, for 5 h [27]. Subsequently, they were depurated ultrasonically in 99.8% isopropyl alcohol at 40 °C for 15 min using an ultrasonic cleaner (Sonorex Super, Bandelin Electronic, Berlin, Germany). All samples were dried using pressed air.

For the calculation of TP and OP values, the Commission Internationale de l’Eclairage (CIE) color data (LabCh) of disc-shaped samples (*n* = 10) were defined using a spectrophotometer (Lovibond Tintometer Model: RT-400) on white (*L* = 0.23, *a* = 0.03, and *b* = − 0.25) and black backgrounds (*L* = 93.85, *a* = − 0.64, and *b* = 2.24). The specular component excluded (SCE) was selected. Data were saved in the spectral range of 400–700 nm at 10-nm intervals.

For the calculation of FL values, the CIE color data (LabCh) of the disc-shaped samples (*n* = 10) were defined using a Minolta CM-3600D spectrophotometer (Konica Minolta Sensing, Inc., Japan) inclusive (UV 100%) and exclusive (UV 0%) the UV ingredient on white (*L* = 0.23, *a* = 0.03, and *b* = − 0.25) background. The SCE was selected. Data were saved in the measurement interval from 360 to 740 nm at a 10-nm pitch.

All measurements were made by the same operator with D65 standard illumination, an observer angle of 10°, and a d/8° measurement geometry [18]. The average values were calculated and recorded. The spectrophotometers were calibrated before each measurement for every sample.

TP, the color difference between readings against a black (*L*_b_, *C*_b_, *H*_b_) and white (*L*_w_, *C*_w_, *H*_w_) background for the same samples was calculated with the CIE2000 formula [28]

$${\text{TP}}_{00} = \sqrt {\left( {\frac{{{\text{LB }}{-}{\text{ LW}}}}{{K_{{\text{L}}} S_{{\text{L}}} }}} \right)^{2} + \left( {\frac{{{\text{CB }}{-}{\text{ CW}}}}{{K_{{\text{C}}} S_{{\text{C}}} }}} \right)^{2} + \left( {\frac{{{\text{HB }}{-}{\text{ HW}}}}{{K_{{\text{H}}} S_{{\text{H}}} }}} \right)^{2} + R_{{\text{T}}} \left( {\frac{{{\text{CB }}{-}{\text{ CW}}}}{{K_{{\text{C}}} S_{{\text{C}}} }}} \right)\left( {\frac{{{\text{HB }}{-}{\text{ HW}}}}{{K_{{\text{H}}} S_{{\text{H}}} }}} \right).}$$

For the OP, the values of the CIE coordinates evaluated on black (*a*_b_, *b*_b_) and white (*a*_w_, *b*_w_) backgrounds of the samples were computed using the behind formula [16, 29]

$${\text{OP }} = \sqrt {\left( {a_{{\text{b}}} - a_{{\text{w}}} } \right)^{2} + \left( {b_{{\text{b}}} - b_{{\text{w}}} } \right)^{2} .}$$

For the FL, the color difference between readings against the UV inclusive (*L*_100_, *C*_100_, *H*_100_) and exclusive (*L*_0_, *C*_0_, *H*_0_) for the same samples was calculated with the CIE2000 formula [28]

$${\text{FL}}_{00} = \sqrt {\left( {\frac{{L_{100} { }{-}{ }L_{0} }}{{K_{{\text{L}}} S_{{\text{L}}} }}} \right)^{2} + \left( {\frac{{C_{100} { }{-}{ }C_{0} }}{{K_{{\text{C}}} S_{{\text{C}}} }}} \right)^{2} + \left( {\frac{{H_{100} { }{-}{ }H_{0} }}{{K_{{\text{H}}} S_{{\text{H}}} }}} \right)^{2} + R_{{\text{T}}} \left( {\frac{{C_{100} { }{-}{ }C_{0} }}{{K_{{\text{C}}} S_{{\text{C}}} }}} \right)\left( {\frac{{H_{100} { }{-}{ }H_{0} }}{{K_{{\text{H}}} S_{{\text{H}}} }}} \right).}$$

*L*, *C*, and *H* show the distinctions in lightness, chroma, and hue in CIEDE2000; RT is the statement for the interplay among chroma and hue distinctions in the blue zone. *S*_L_, *S*_C_, and *S*_H_ are weighting tasks that set the all-color distinction for variety in place of the color distinction in *L*, *a*, and *b* coordinates. The parameterized determinants *K*_L_, *K*_C_, and *K*_H_ are correcting expressions for variation in experimental situations. In this study, the parameterized determinants of the CIEDE2000 color distinction formula were adjusted to 1 [28].

**Fig. 1:**
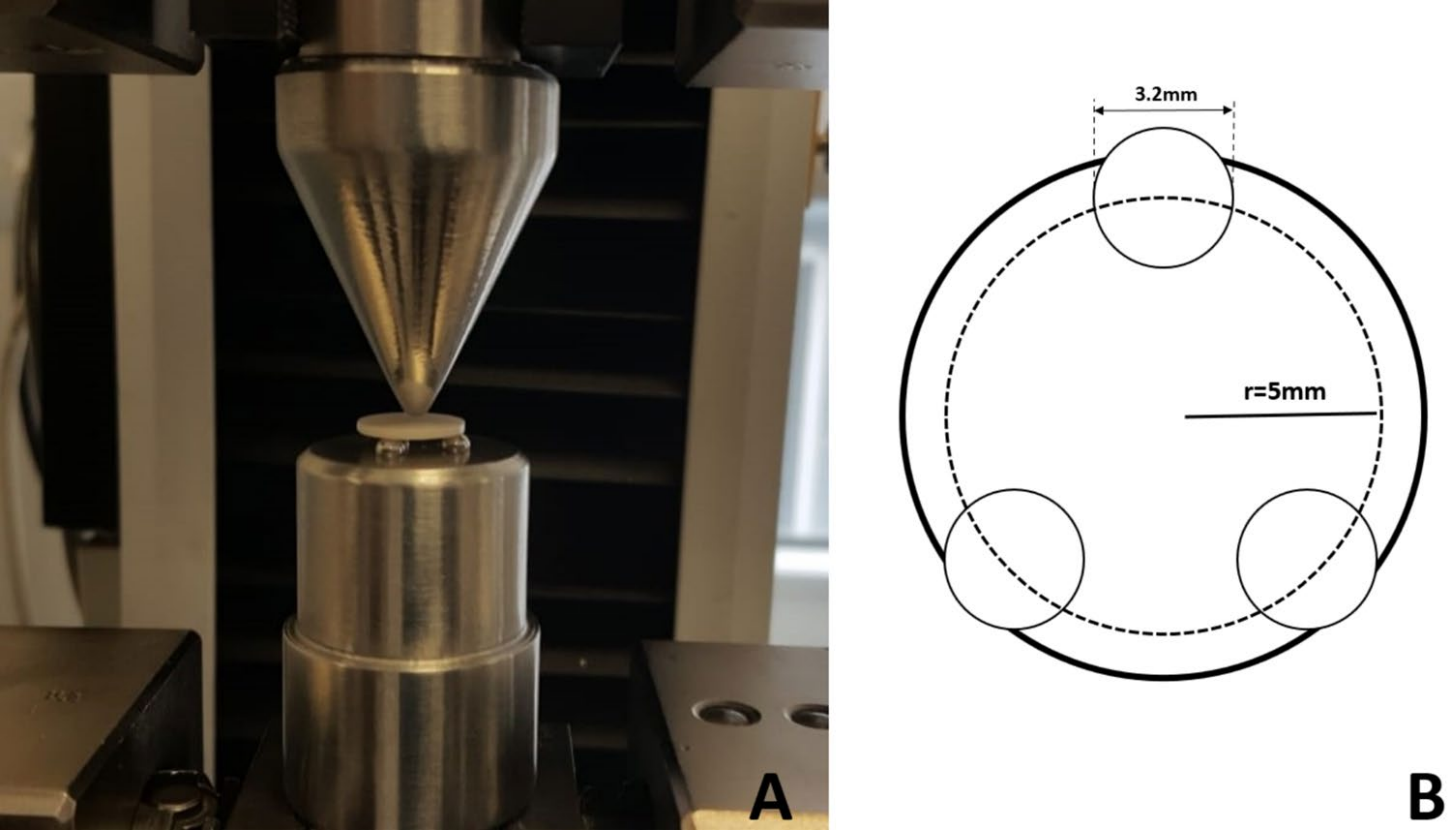
Biaxial breaking strength test. **A** Crushing mechanism. **B** Schematic representation

BFS, biaxial flexural strength (MPa); *P*, fracture force (N); *d*, sample thickness (mm). Additionally, *X* and *Y* were calculated using the following formulas:

Color measurements completed disc-shaped samples (*n* = 10) were tested to BFS testing in an Instron universal testing machine model 3345 (MA, USA) (Fig. 1). For the fracture test, three steel balls, 3.2 mm in diameter were emplaced at equal distances on a 10 mm diameter (*r*1) circle on a metal platform (Fig. 1). Each sample was positioned to align with the steel balls, and a 1.4-mm-diameter (*r*2) steel piston applied force at a speed of 0.5 mm/min. The force at which the sample broke (*P*) was saved in Newtons (N). The BFS of each sample was computed using the following formula [3, 8]:

$${\text{BFS }} = \left[ { - 0,2387P\left( {X - Y} \right)} \right]/d2;$$

$$X = \left( {1 + \upsilon } \right) \, \ln \left( {r2 \, /r3} \right)2 \, + \, \left[ {\left( {1 - \, \upsilon } \right) \, /\left( {r2 \, / \, r3} \right)2} \right]$$

$$Y = \left( {1 + \upsilon } \right) \, \left[ {1 + \, \ln \left( {r1 \, /r3} \right)2} \right] + \, \left( {1 - \upsilon } \right) \, \left( {r1 \, /r3} \right)2.$$

*υ*, Poisson ratio of Zirconia (0.25) [30]; *r*1, radius of the support circle (mm); *r*2, radius of the piston (mm); *r*3, radius of the sample (mm)^2^.

MZ square-shaped samples from each group were polished using 400, 600, 800, 1200, 1600, and 2000‑grit papers (Struers, OH, USA), respectively. The chemical content (wt%) of the sample from each sintering group was determined by WD-XRF, using a PANalytical model AXIOS XRF wavelength-dispersive spectrometer, operating at 60 kV and 50 mA.

After WD‑XRF analysis, the same MZ square-shaped samples were thermally etched for 20 min at 1250 °C to identify grain boundaries. To prevent considerable changes in GS, a rapid heating rate (40 °C/min) and a low heat temperature were performed. After each MZ square-shaped sample was coated with gold/palladium, scanning electron microscope (FE-SEM; Gemini 500, Carl Zeiss AG, Germany) images were taken at 20,000 × magnification for GS. The average GS for each sample was calculated by measuring at least 200 grains across ten varied spots on FE-SEM micrographs, using software (Image J, NIH) based on the linear intercept method [31].

One MZ square-shaped sample remaining from each group was embedded in resin (ProbleMet Powder; Buehler Co.) and polished to using 240–2500 g of waterproof abrasive and 0.02-µm colloidal silica suspension. It was then ultrasonically depurated using deionized water and ethanol. Subsequently, X-ray diffraction (XRD) patterns of square-shaped samples were recorded (Empyrean; Malvern Panalytical Ltd.) using Cu-Kα radiation (*λ* = 1.54 Å) in the 2*θ* angle scanning interval of 20°–90° (40 mA, 45 kV generator adjustments, 0.026 step size). Mineral content and crystal structures of square samples were obtained and characterized according to the International Center for Diffraction Data (ICDD) using XRD patterns in a computer program (HighScore; Malvern Panalytical Ltd.).

All data were analyzed by a statistical analysis program (SPSS 22.0, USA), with a significance grade set at *α* = 0.05. The conformity of the data with the normal distribution was assessed with the Shapiro–Wilk. Among the dependent variables, only grain size was analyzed with the Mann–Whitney *U* test, because it did not comply with the normal distribution. Since all other dependent variables exhibited a normal distribution, they were analyzed by an independent sample *t* test.

## Results

The mean and standard deviation values for TP_00_, OP, FL_00_, fracture strength, and GS for both standard and fast sintering procedures are presented in Tables 4 and 5. The sintering procedure had a statistically significant effect on TP_00_, OP, fracture strength, and GS (*P* < 0.05), while it did not have a statistically significant effect on FL_00_ (*P* > 0.05).

**Table 4:** Mean and standard deviation values of the investigated parameters

Parameters investigated	*n*	Sintering procedures		Mean difference	*P*
		Standard	Fast		
TP_00_	10	7.87 ± 0.42	7.21 ± 0.72	0.66	0.025
OP	10	6.61 ± 0.36	7.22 ± 0.28	0.61	0.001
FL_00_	10	0.23 ± 0.11	0.18 ± 0.10	0.05	0.277
Fracture strength	10	1597.93 ± 90.44	1336.54 ± 114.96	261	0.000

**Table 5:** Values of the grain size parameter

Sintering procedures	Mean	Std. error	Median	Minimum	Maximum	Range	Interquartile range	*P*
Standard	311.19	7.05	306.68	84.77	793.63	708.86	158.64	0.000
Fast	355.03	9.16	351.88	102.33	718.26	615.93	212.62	

### Optical properties’ results

**Fig. 2:**
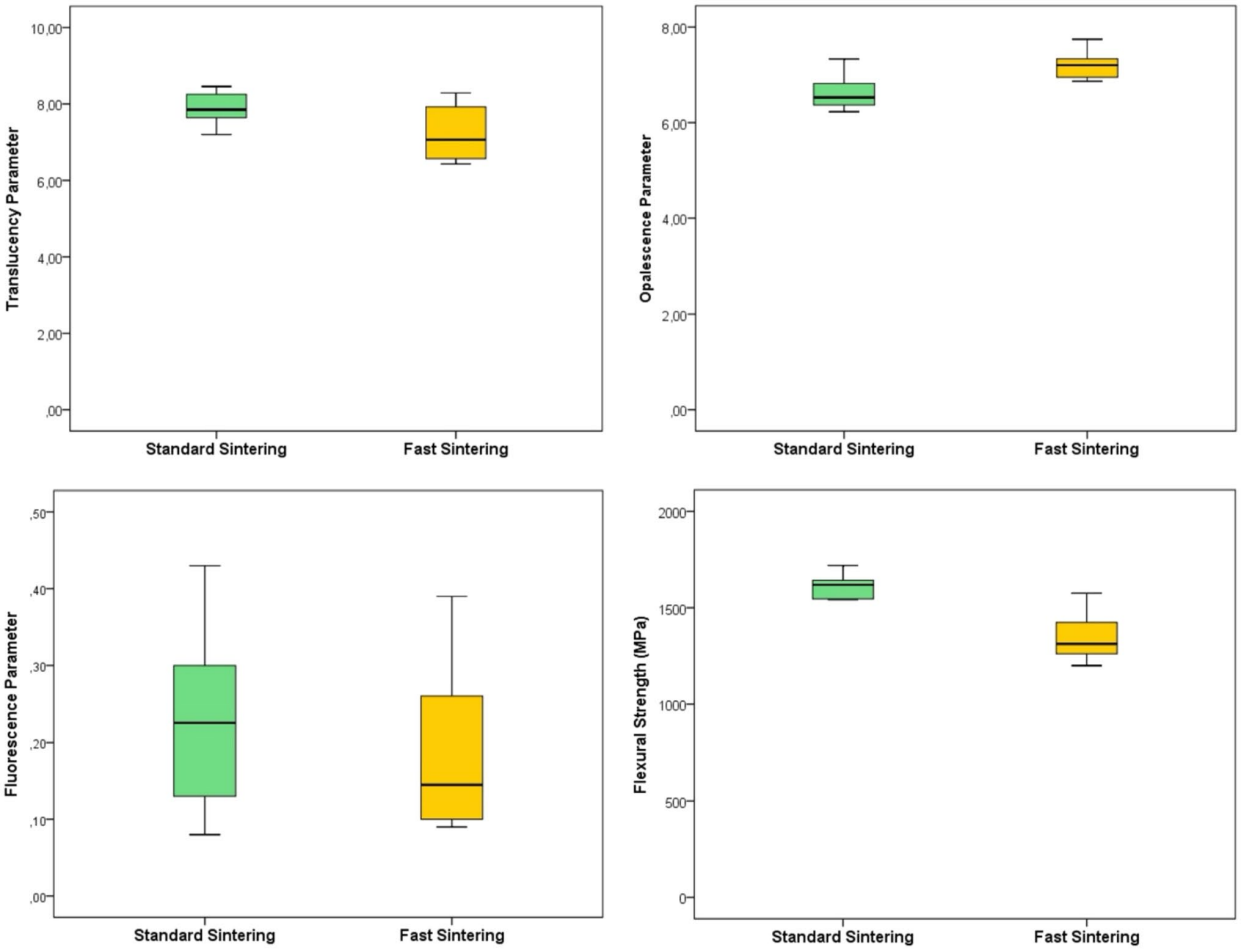
Comparison of translucency, opalescence, fluorescence, and fracture strength between standard and fast sintering procedures

The TP_00_ values showed a significant difference between the two sintering procedures (*P* = 0.025), with the standard sintering group exhibiting higher translucency values (7.87 ± 0.42) compared to the fast sintering group (7.21 ± 0.72) (Table 4 and Fig. 2). The OP values also varied significantly (*P* = 0.001), with the fast sintering group displaying higher OP values (7.22 ± 0.28) than the standard sintering group (6.61 ± 0.36). However, FL_00_ values between the groups were not significantly different (*P* = 0.277), with the standard and fast sintering groups showing similar fluorescence values (0.23 ± 0.11 and 0.18 ± 0.10, respectively).

### Fracture strength results

The BFS values of both groups were significantly different (*P* = 0.000). The standard sintering group exhibited a mean fracture strength of 1597.93 ± 90.44 MPa, whereas the fast sintering group showed a lower mean fracture strength of 1336.54 ± 114.96 MPa (Table 4 and Fig. 2). Despite the reduction in fracture strength with fast sintering, all measured values remained above the clinically acceptable threshold of 900 MPa, as recommended by ISO 6872 standards.

### Grain size and microstructural analysis results

**Fig. 3:**
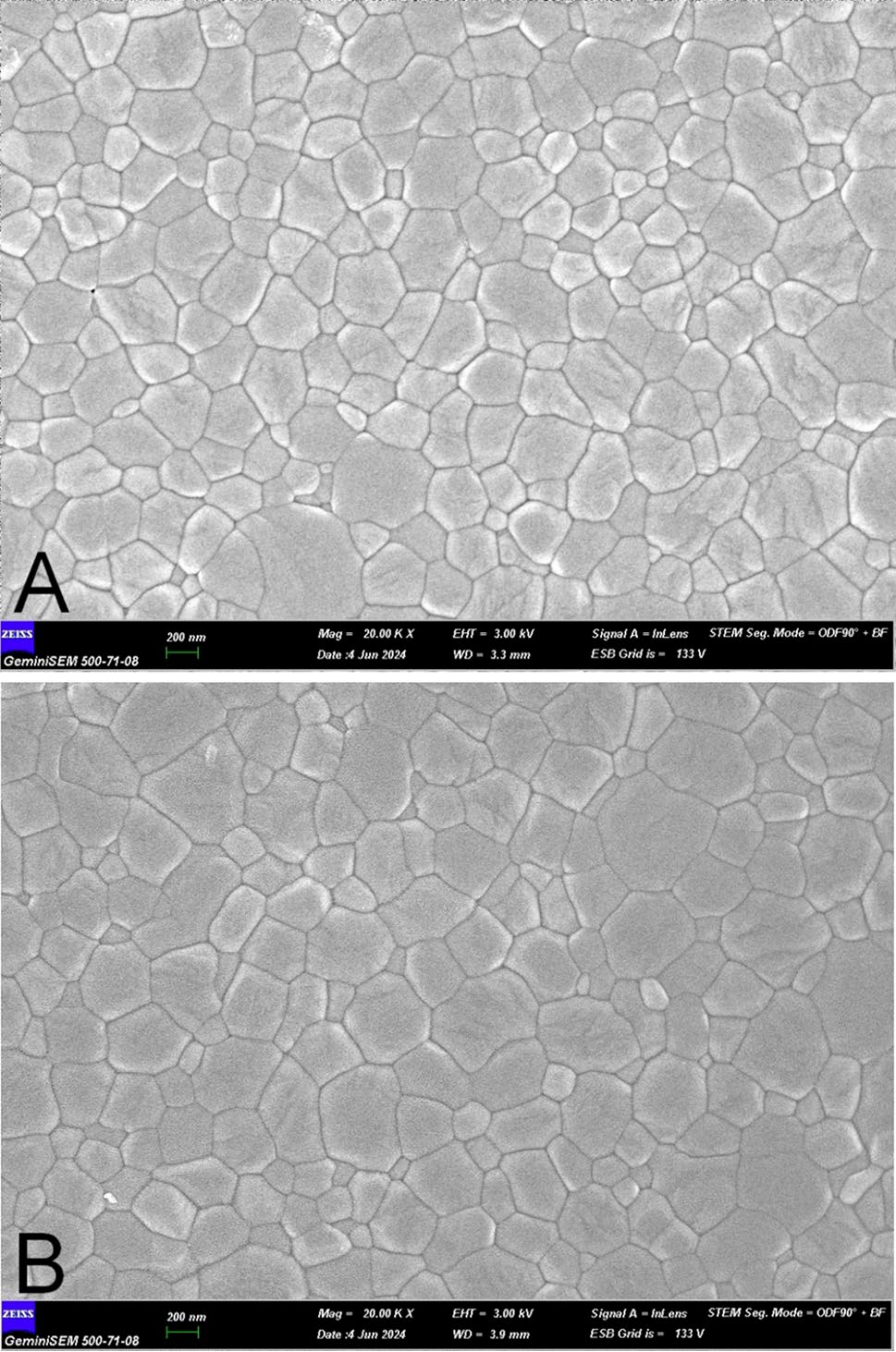
FE-SEM micrographs obtained at × 20,000 magnification. **A** standard-sintered sample. **B** Fast-sintered sample

The FE-SEM micrographs (Fig. 3) illustrate the grain morphology of both sintering groups. A statistically significant increase in GS was observed in the fast sintering group compared to the standard sintering group (*P* = 0.000) (Table 5). The mean GS was 311.19 ± 7.05 nm for the standard sintering group and 355.03 ± 9.16 nm for the fast sintering group. The grain distribution appeared more uniform in the standard sintering group, while the fast sintering process led to relatively larger grains with increased variability in size.

### X-ray diffraction and chemical composition results

**Fig. 4:**
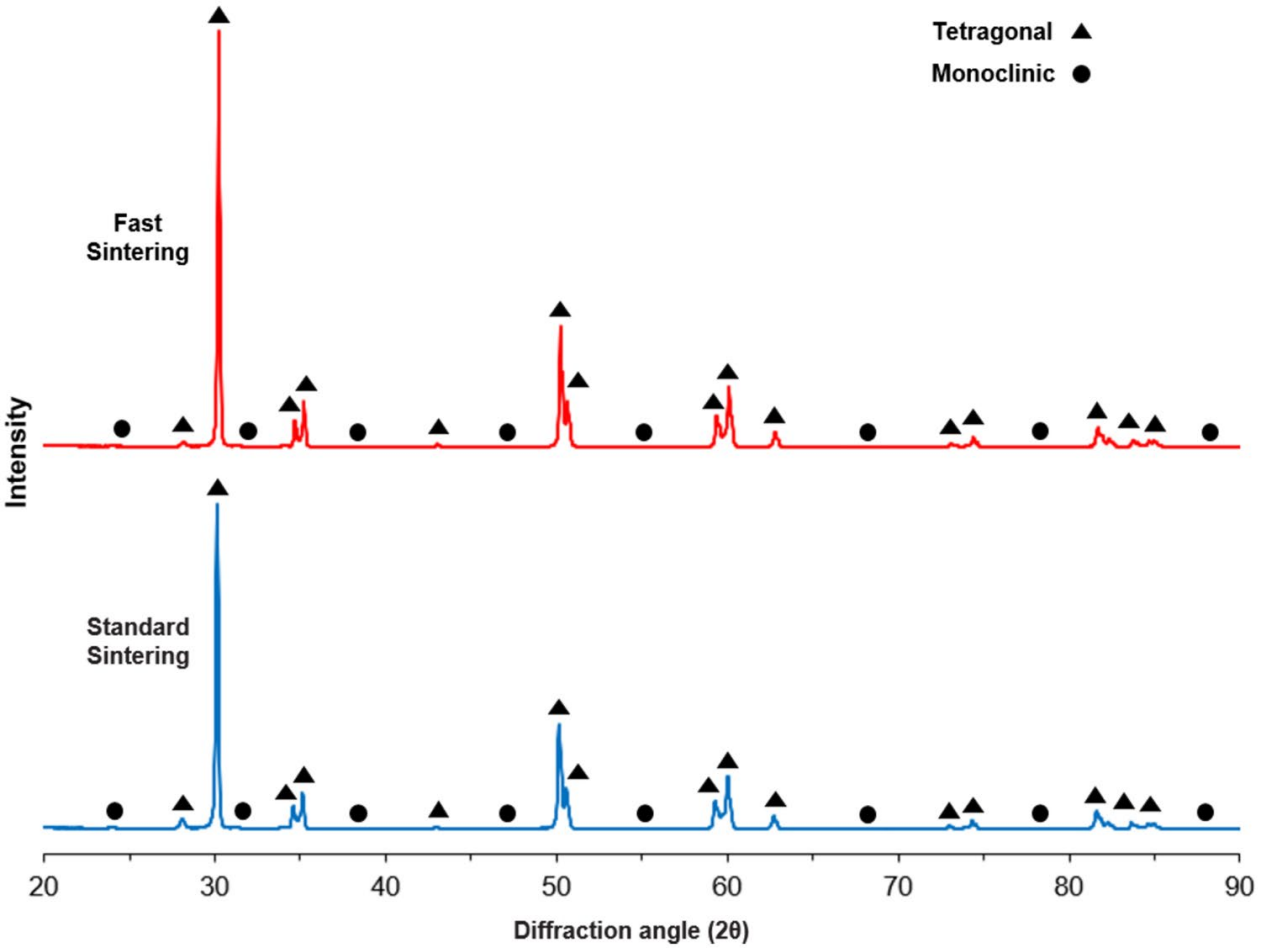
Radiograph diffractometer patterns. Blue shows standard sintering and red shows fast sintering

XRD patterns indicated that both sintering procedures yielded similar diffraction peaks, with the presence of tetragonal and monoclinic phases in both groups (Fig. 4). The phase composition analysis showed that the standard sintering group contained zirconium oxide (tetragonal and monoclinic) and yttrium zirconium oxide (tetragonal). The fast sintering group also exhibited zirconium oxide (tetragonal and monoclinic), but with an additional presence of hafnium oxide (monoclinic) (Table 6).

**Table 6:** Mineral analysis and crystal structure content after sintering procedures

Sintering procedures	Compound name	ICDD reference code	Crystal system	Chemical formula
Standard	Zirconium oxide	01-072-2743	Tetragonal	ZrO_2_
	Yttrium zirconium oxide	04-016-2090	Tetragonal	Y_0.127_Zr_0.873_O_1.937_
	Zirconium oxide	04-011-8814	Monoclinic	ZrO_2_
Fast	Zirconium oxide	01-075-9646	Tetragonal	ZrO_2_
	Zirconium oxide	01-078-0047	Monoclinic	ZrO_2_
	Hafnium oxide	04-014-7409	Monoclinic	HfO_2_

*ICDD* International Centre for Diffraction Data

The chemical composition analysis revealed minor differences in oxide weight percentages between the two sintering procedures (Table 7). The fast sintering group exhibited a slightly lower ZrO_2_ content (88.76%) compared to the standard sintering group (92.03%), while HfO_2_ (2.11%) and Al_2_O_3_ (0.40%) levels were higher in the fast sintering group than in the standard sintering group (1.47% and 0.19%, respectively).

**Table 7:** Chemical analysis (weight %) of the different sintered samples

Sintering procedures	Oxide percentage by weight (weight %)			
	ZrO_2_	Y_2_O_3_	HfO_2_	Al_2_O_3_
Standard	92.03	6.02	1.47	0.19
Fast	88.76	5.98	2.11	0.40

## Discussion

In this study, different sintering procedures affected TP_00_, OP, fracture strength, and GS statistically significantly but did not affect FL_00_ statistically significantly (Tables 4, 5). For these reasons, the null hypothesis was rejected for all parameters except the fluorescence parameter.

Monolithic zirconia restorations were introduced as an alternative treatment option with excellent mechanical properties, eliminating the need for any veneering ceramic. Nonaka et al. reported that although the BFS values of fast sintering were lower than those of standard sintering, they were above the clinically acceptable threshold of 900 Mpa [8]. This result is compatible with the present study. Dateraksa et al. [30] noted that samples sintered with a lower heating rate exhibited higher fracture strength values. In the present study, the group S had a temperature heating rate of 10 °C/min, while the group F had 25 °C/min, which is coherent with the study by Dateraksa et al. [30] Durkan et al. [32] reported lower BFS values than the present study. In the present study, the holding time at the final sintering temperature for group F was 30 min, while it was 120 min for group S. In Durkan et al.'s study [32], this value was at most 180 min. The difference in the present study results could be related to the shorter holding time at the higher final sintering temperature compared to that of Durkan and colleagues. An increase in the holding time at the final sintering temperature might decrease BFS values in Durkan and colleagues' study. Further studies involving different sintering procedures are needed. For brittle dental materials like full ceramics, fracture strength is a mechanical test used to predict clinical performance. Considering the fracture strength values, all samples in groups S and F exhibited mean BFS values greater than 900 MPa, which recommended by ISO 6872 standards [8], making them suitable for monolithic restorations with four or more units in both the anterior and posterior regions. Consistent with the study of Cokic et al. [10], in this study, it was found that the GS of standard-sintered MZ samples was significantly smaller than the GS of fast-sintered MZ samples (Table 5). In the previous studies, it was reported that fracture strength increased when the GS of MZ decreased [32, 33] which is in consistent with those of the present study.

Studies evaluating the effect of 3Y-TZP monolithic zirconium sinterization procedure on TP are limited. In this study, the TP values of standard-sintered samples were higher than those of fast-sintered samples (*P* = 0.025) (Table 4). This is in consistent with those of the most studies comparing standard and fast sintering procedures for 3Y-TZP samples [18, 22]. Inversely to this study, Liu et al. [34] discovered that the translucency was not significantly affected by the sintering programs (*P* > 0.05). This discrepancy could be associated with distinctions in the compositional structure of the MZ samples used in Liu et al.’s study. Liu et al. used 4, 5, and 6Y-TZP monolithic zirconia, while 3Y-TZP monolithic zirconia was used in the present study. Salas et al. [35] showed that the translucency clinically perceptibility threshold is 1.33 units, whereas 4.43 units is the translucency clinically acceptability threshold. In this study, the mean TP difference (Table 4) between fast and standard sintering was lower than the perceptibility threshold level. This is in consistent with those of previous studies [18, 22]. For this reason, the influence of the sintering process on TP is not clinically perceivable to the human eye. It has been reported that with an increase in GS, the grain boundaries decrease, resulting in an increase in the translucency of zirconia [3, 33]. However, contrary to what was expected, the average TP value of the fast-sintered samples with a higher average GS was lower than the standard-sintered samples in this study (Tables 4, 5). At the same time, according to the results of this study, the percentage of Al_2_O_3_ and HfO_2_ of the fast-sintered samples was higher than the standard-sintered samples (Table 7). In determining the optical properties of MZ, not only the boundaries of the grains but also the structure content of the grains are important [36]. In the previous studies, it has been shown that TP decreased when the Al_2_O_3_ percentage of MZ increased [36]. Based on this information, the higher percentage of Al_2_O_3_ and HfO_2_ in the fast-sintered samples compared to the standard-sintered samples in this study may explain the decrease in TP.

Opalescence is an important optical property that makes the natural tooth look alive [37]. If the OP value is between 4 and 9, opalescence is barely perceivable with the human eye; if it is below 4, it is not perceivable [38]. In the present study, both sintering procedures resulted in barely perceivable OP values in the samples (6.61 ± 0.36 and 7.22 ± 0.28). The mean OP difference between fast and standard sintering was 0.61 (Table 4). The authors of this study think that this difference may be considered clinically negligible. Ardu et al. reported that the OP values of the enamel–dentin complex and the enamel layer were 4.8 and 7.4, respectively [13]. It can be said that the OP values measured in this study are sufficient to mimic natural human teeth. In studies where OP values are analyzed, the values found were variable. The OP values in the Soult et al. study [14] (8.9–10.1) were higher than the present study, whereas the OP values in the studies of Gülnal et al. [39] (4.35–4.39) and Juntavee et al. [15] (1.25–2.83) were lower than the present study. These differences may be because of the varied brands of zirconia blocks used. Further studies using different brands of blocks are needed to research the effect of various sintering processes on the opalescence of zirconia.

Natural teeth spread fluorescence under the influence of UV light [40]. This feature ensures that the tooth has a whiter and brighter character and gives vitality to the natural tooth. Aesthetic restorative materials must have sufficient fluorescence to give the restoration a natural appearance in different light conditions [41]. The dentin of human teeth is more fluorescent than the enamel, and most of the fluorescence in human teeth originates from the dentin [40]. Lee et al. submitted that the FL rate of human dentin was 0.73 ± 0.04 [42]. In this study, the FL values of all samples (0.23 ± 0.11 and 0.18 ± 0.10) were lower than those of human dentin. The fluorescence property of the zirconia block used in this study is insufficient to mimic natural teeth. In the present study, no glaze was performed to the samples. To achieve fluorescent properties closer to the natural tooth, glazing materials with fluorescent properties can be used [43]. In Albayrak et al.'s study [21] on the effect of varied sintering procedures on fluorescence, contrary to this study, they found a statistically significant higher fluorescence value in fast sintering (2.81 ± 2.28) than in standard sintering (1.1 ± 0.61). This difference may be due to the different MZ block brand they used.

Based on the results of this study, tetragonal phase structures were found in strong peaks and monoclinic phase structures were found in weak peaks in both sintering procedures. In agreement with the previously reported results [34, 44], fast sintering of samples in this study did not cause a change in the phases in the strong and weak peaks, and the XRD patterns were quite similar. No cubic phase was detected in the MZ samples in this study, consistent with the previous studies [45, 46]. Studies by Liu et al. and Alshahrani et al. showed that cubic phases in addition to tetragonal phases were detected in the strong peaks [34, 44]. This may be due to the different MZ blocks used and the different sintering times and temperatures.

MZ restorations are subjected to the fluids of the oral cavity, which lead to hydrothermal aging [27]. The optical [27] and mechanical properties of MZ restorations may be affected by hydrothermal aging. Studies have indicated that aging for 5 h at 134 °C, 0.2 MPa pressure in an autoclave is equivalent to 15–20 years [47] or 5 years [27] of clinical use. In this study, to simulate clinical usage, all samples were autoclaved at 134 °C and pressurized to 0.2 MPa for 5 h. This study has some limitations that should be acknowledged. First, the research was conducted in vitro, which may not fully replicate the complex conditions of the oral environment, including temperature fluctuations, mechanical loading, and chemical interactions with saliva and food. Second, the study did not utilize anatomically shaped fixed prostheses that reflect the morphology of natural teeth. Additionally, only a single brand of zirconia block was tested, and variations in chemical composition among different manufacturers could influence the optical and mechanical properties. Finally, the absence of coloration, surface treatments, and clinical adjustments may have limited the study’s ability to fully evaluate the aesthetic and functional aspects of the materials. Future studies should consider multiple zirconia brands and clinical factors to validate these findings on anatomically shaped samples.

## Conclusion

Within the limitations of this study, the following conclusions have been reached:

Fast sintering reduces the fracture strength of MZ, but the values were all above the clinically acceptable level according to ISO 6872 standards. Fast sintering had no clinically significant effect on optical properties of MZ. Fast sintering affected the GS of MZ statistically significantly, while crystal structure and chemical content percentage were affected limitedly. Considering the time and cost, the fast sintering procedure of MZ can be recommended.

## Data Availability

The data that support the findings of this study are available from the corresponding author upon reasonable request.
